# Reliability and quality of cognitive impairment educational content on Douyin and Bilibili: A cross-sectional content analysis

**DOI:** 10.1097/MD.0000000000048941

**Published:** 2026-05-22

**Authors:** Yuzhang Liang, Yifeng Xie, Haiyan Song, Xiaoxuan Fan, Yike Liu, Ya Na, Jiamin Gao, Ying Ao, Chunyan Chen

**Affiliations:** a Affiliated Bayannur Clinical College, Inner Mongolia Medical University, Bayannur, China; b Department of Neurology, Affiliated Bayannur Clinical College, Inner Mongolia Medical University, Bayannur, China.

**Keywords:** Bilibili, cognitive impairment, Douyin, information quality, short video, TikTok

## Abstract

This study aimed to evaluate the content characteristics, quality, and reliability of cognitive impairment educational videos on Douyin and Bilibili and examine whether video duration and user engagement are associated with information quality. This cross-sectional content analysis searched the Chinese domestic version of TikTok (Douyin; https://www.douyin.com/) and Bilibili (https://www.bilibili.com/) through their official web interfaces on March 12, 2026. Searches were performed in logged-out mode using the keyword “cognitive impairment” via a desktop browser. The first 150 results from each platform were screened, and 250 eligible videos were included (133 from Douyin and 117 from Bilibili). Video quality and reliability were evaluated with the Global Quality Score, modified DISCERN, Journal of the American Medical Association benchmark criteria, and Video Information and Quality Index. Overall educational quality was moderate. Across both platforms, content focused primarily on clinical manifestations and treatment, whereas epidemiology, diagnosis, and prognosis were insufficiently covered. Douyin videos had significantly higher Global Quality Score, modified DISCERN, Journal of the American Medical Association, and Video Information and Quality Index scores than Bilibili videos (all *P* < .001), despite being substantially shorter. Videos uploaded by doctors or other health professionals showed the highest quality and reliability, whereas videos uploaded by individual users generated stronger engagement. Correlations between engagement indicators and quality scores were weak, indicating that popularity did not reliably reflect educational value. Cognitive impairment videos on Douyin and Bilibili showed substantial variability in quality and incomplete content coverage. Professional participation, clearer source disclosure, and platform-level governance may improve the accuracy and practical utility of short-video health education on cognitive impairment.

## 1. Introduction

Cognitive impairment is a neurological syndrome characterized by a decline in memory, executive function, language, and activities of daily living. It can progress to disability, dependence on care, and death, and it has become a major public health concern in aging societies.^[1,2]^ Early public recognition of cognitive impairment may facilitate timely help-seeking, earlier diagnosis, and more appropriate long-term management.^[3,4]^

Social media platforms have become important channels for health communication because of their large user base, rapid dissemination, and strong interactivity.^[5,6]^ However, the same features that make social media attractive also increase the risk that incomplete, inaccurate, or weakly sourced health information will spread widely.^[7]^ Previous studies have shown that short-video content related to dementia, diabetes, thyroid cancer, gastric cancer, and other clinical topics often receives substantial engagement while showing uneven scientific quality and reliability.^[8,9]^

Although videos about cognitive impairment are increasingly available on Chinese video platforms, comparative evidence regarding their content structure, educational value, and reliability remains limited.^[9,10]^ In particular, the Chinese domestic version of TikTok (Douyin) and Bilibili differ in content ecology, video length norms, and audience interaction patterns, which may influence both dissemination and information quality. Therefore, this study conducted a cross-sectional content analysis of cognitive impairment videos on Douyin and Bilibili to compare platform characteristics, uploader composition, thematic coverage, and quality and reliability scores, and to examine whether video duration and engagement metrics were associated with educational quality.

## 2. Methods

### 2.1. Study design

This was a cross-sectional content analysis of publicly accessible educational videos related to cognitive impairment. The study aimed to assess content characteristics, dissemination performance, information quality, and reliability across 2 major Chinese video platforms.

### 2.2. Data sources and search strategy

**Figure 1.**
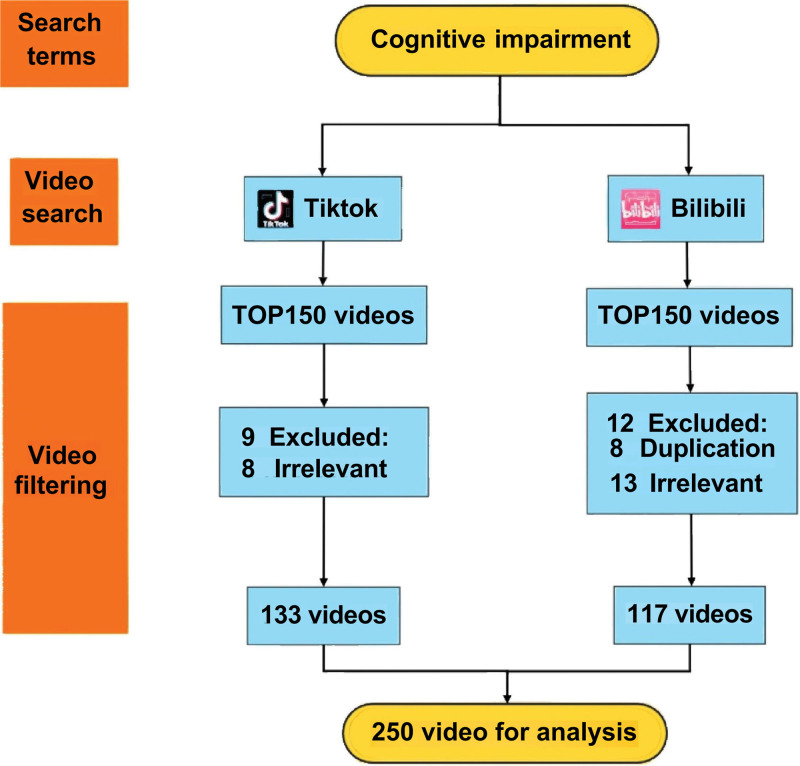
Flowchart of video search and selection. Videos related to cognitive impairment were searched on TikTok and Bilibili.

Data were collected on March 12, 2026, through the official web interfaces of Douyin (the Chinese domestic version of TikTok; https://www.douyin.com/) and Bilibili (https://www.bilibili.com/). Searches were performed using a desktop browser in logged-out mode to reduce the influence of personalized recommendations and prior browsing history. The search keyword was “cognitive impairment.” The first 150 videos returned by each platform search engine were collected as the initial sample. Videos were screened on the same day, and those that met the eligibility criteria were retained for analysis. After screening, 250 videos were included: 133 from Douyin and 117 from Bilibili (Fig. 1).

## 3. Eligibility criteria

Videos were eligible if they were directly related to cognitive impairment, could be played normally, contained complete audiovisual content, and were publicly accessible during the study period. Videos were excluded if they were duplicates, unrelated to cognitive impairment, advertisements or purely commercial promotions, clearly lacked educational content, or could not be fully assessed because they had been removed, deleted, or restricted.

### 3.1. Data extraction

For each included video, the following variables were extracted: platform source, video duration, number of likes, collections, comments, and shares, uploader type, thematic content coverage, and quality and reliability scores. Uploaders were classified as doctors or other health professionals (DHPs), non-doctor health professionals or health-related organizations, and individual users (IUs). Content was categorized into 6 predefined domains: epidemiology, etiology, clinical manifestations, diagnosis, treatment, and prognosis. Each domain was coded as not covered, partially explained, or fully explained.

### 3.2. Quality and reliability assessment

Video quality and reliability were evaluated using 4 commonly used instruments: Global Quality Score (GQS),^[11]^ modified DISCERN (mDISCERN),^[12]^ Journal of the American Medical Association (JAMA) benchmark criteria,^[13]^ and Video Information and Quality Index (VIQI).^[14]^ Because these tools were originally developed for broader web-based or video-based health information rather than short-video platforms, we predefined short-video-oriented operational rules before formal scoring. For GQS, raters focused on overall educational usefulness, logical flow, and completeness within the constraints of the platform-specific video format. For mDISCERN, source citation, balance, mention of uncertainty, and referral to additional information were judged based on spoken content, on-screen text, captions, account identity, and the video description when available. For JAMA, authorship, attribution, currency, and disclosure were scored only when the corresponding information was explicitly visible within the video, account page, or directly linked description. For VIQI, information flow, accuracy, precision, and production quality were scored while recognizing short-video features such as subtitles, animations, screenshots, interview clips, and title-content consistency. Detailed scoring criteria are provided in Tables S1 to S4.

### 3.3. Rater procedure

Two senior chief neurologists independently completed data extraction and quality scoring after pilot calibration on a subset of videos. Disagreements were resolved through discussion, and persistent discrepancies were adjudicated by a third senior chief neurologist. This procedure was used to improve scoring consistency and methodological transparency.

## 4. Statistical analysis

Descriptive statistics were used to summarize video characteristics. Normally distributed continuous variables are presented as mean (standard deviation), non-normally distributed continuous variables as median (interquartile range), and categorical variables as frequency (%). Between-platform comparisons were performed using the independent-samples *t* test for normally distributed variables and the Mann–Whitney *U* test for non-normally distributed variables. Comparisons among 3 uploader groups were conducted using the Kruskal–Wallis *H* test, followed by Dunn post hoc tests when the overall comparison was significant. Spearman rank correlation coefficients were used to evaluate associations among video duration, engagement metrics, and quality and reliability scores. All tests were two-sided, and *P* < .05 was considered statistically significant. Analyses were performed using R software (version 4.3.2; R Foundation for Statistical Computing, Vienna, Austria).

## 5. Ethics

This study analyzed only publicly accessible online videos and did not involve intervention, interaction with human participants, patient data, or identifiable personal information. Therefore, formal ethics committee or institutional review board approval was not required, and informed consent was not applicable.

## 6. Results

### 6.1. Video screening and overall characteristics

**Figure 2.**
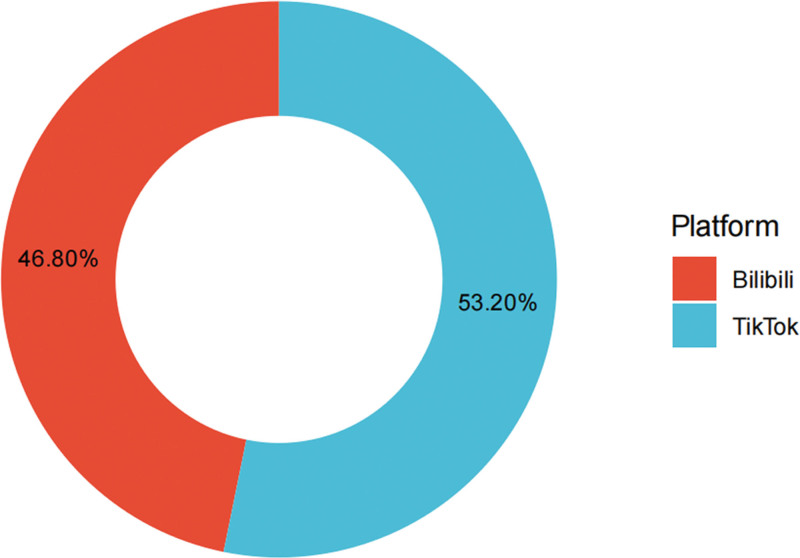
Distribution of included videos by platform. Among the 250 included videos, 53.2% were from TikTok and 46.8% were from Bilibili.

A total of 250 videos were included, of which 133 (53.2%) were from Douyin, and 117 (46.8%) were from Bilibili. The overall median video duration was 180.50 seconds (interquartile range: 76.25–555.00). Median engagement counts were 119.50 for likes, 62.00 for collections, 8.00 for comments, and 31.50 for shares. The median GQS, mDISCERN, JAMA, and VIQI scores were 3.00, 3.00, 2.00, and 11.00, respectively, indicating a moderate overall level of educational quality and reliability (Table 1; Fig. 2).

**Table 1:** Global quality score criteria.

Description	Score
Poor quality; poor flow of the videos; most information missing; not at all useful for patients	1
Generally poor quality; some information listed, but many important topics missing; of very limited use to patients	2
Moderate quality; suboptimal flow; some important adequately discussed, but other information poorly discussed; somewhat useful for patients	3
Good quality and generally good flow; most of the relevant information listed, but some topics not covered; useful for patients	4
Excellent quality and flow; very useful for patients	5

### 6.2. Uploader characteristics and platform distribution

**Figure 3.**
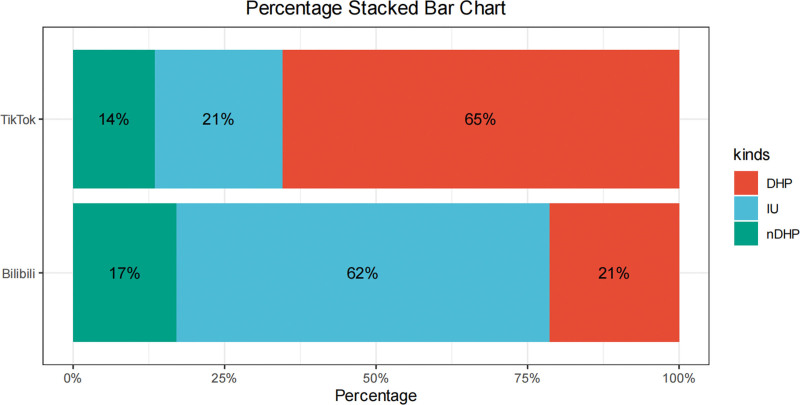
Distribution of uploader types across TikTok and Bilibili. DHPs = doctors or other health professionals, IUs = individual users, NDHPs = non-doctor health professionals or health-related organizations.

Bilibili videos were substantially longer than Douyin videos (median 590.00 vs 88.00 seconds, *P* < .001) and received significantly more collections and shares (both *P* < .001), whereas likes and comments did not differ significantly between platforms (Table 2). In terms of uploader composition, Douyin was dominated by DHP accounts, whereas Bilibili was dominated by IUs (Fig. 3). When stratified by uploader type, IU videos were the longest and generated the highest likes, collections, comments, and shares, whereas DHP videos showed more limited dissemination but better educational quality and reliability (Table 3).

**Table 2:** Modified DISCERN criteria.

Reliability score
1. Is the video clear, concise, and understandable?
2. Are valid sources cited?
3. Is the content presented balanced and unbiased?
4. Are additional sources of content listed for patient reference?
5. Are areas of uncertainty mentioned?

**Table 3:** JAMA benchmark criteria for online health information.

Score	Score component	
1	Authorship	Author and contributor credentials and their affiliations should be provided.
1	Attribution	Clearly lists all copyright information and states references and sources for content.
1	Currency	Initial date of posted content and subsequent updates to content should be provided.
1	Disclosure	Conflicts of interest, funding, sponsorship, advertising, support, and video ownership should be fully disclosed.

JAMA = Journal of the American Medical Association.

### 6.3. Content analysis

**Figure 4.**
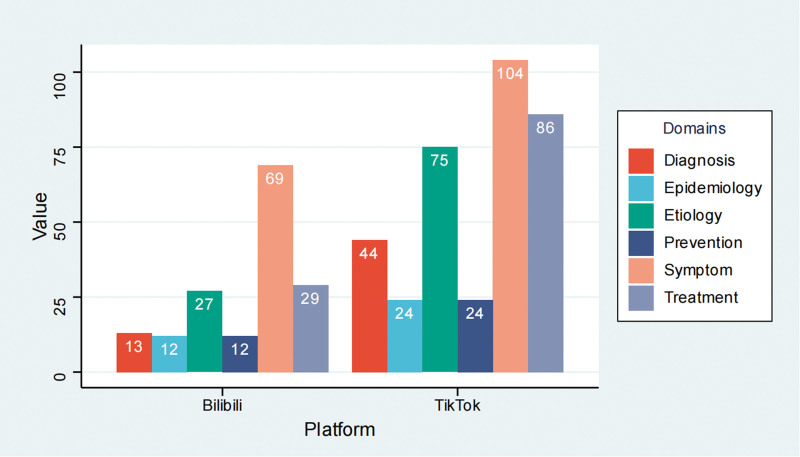
Comparison of content domain coverage between TikTok and Bilibili. The figure shows the number of videos covering each predefined content domain, including diagnosis, epidemiology, etiology, prevention, symptoms, and treatment, across the 2 platforms.

Across both platforms, the most commonly covered domains were clinical manifestations and treatment. Clinical manifestations had the highest overall coverage, with 69 of 250 videos (27.6%) fully explaining this domain and only 77 (30.8%) not covering it. Treatment was the second most frequently addressed domain. In contrast, epidemiology and prognosis were markedly underrepresented: 214 videos (85.6%) did not address either topic, and no video fully explained either domain. Diagnosis and etiology were also insufficiently covered (Table 4). The general content pattern was similar across platforms, with stronger emphasis on symptoms and treatment than on epidemiology, diagnosis, and prognosis (Fig. 4).

**Table 4:** Video information and quality index criteria.

Domain	Description
Information flow	Assesses whether the video presents information coherently and logically, in a reasonable order, and is easy for viewers to follow.
Information accuracy	Assesses whether the information is accurate, evidence-based, and supported by authoritative sources, and whether the content contains errors or is misleading.
Video quality	Assesses production and presentation quality, including the use of supportive elements (e.g., images, animations, interviews, captions/subtitles, and summaries). Each element contributes 1 point (maximum 5 points).
Precision	Assesses consistency between the video title and content (i.e., whether the content matches what is promised by the title and whether discrepancies are present).

### 6.4. Quality and reliability by platform

**Figure 5.**
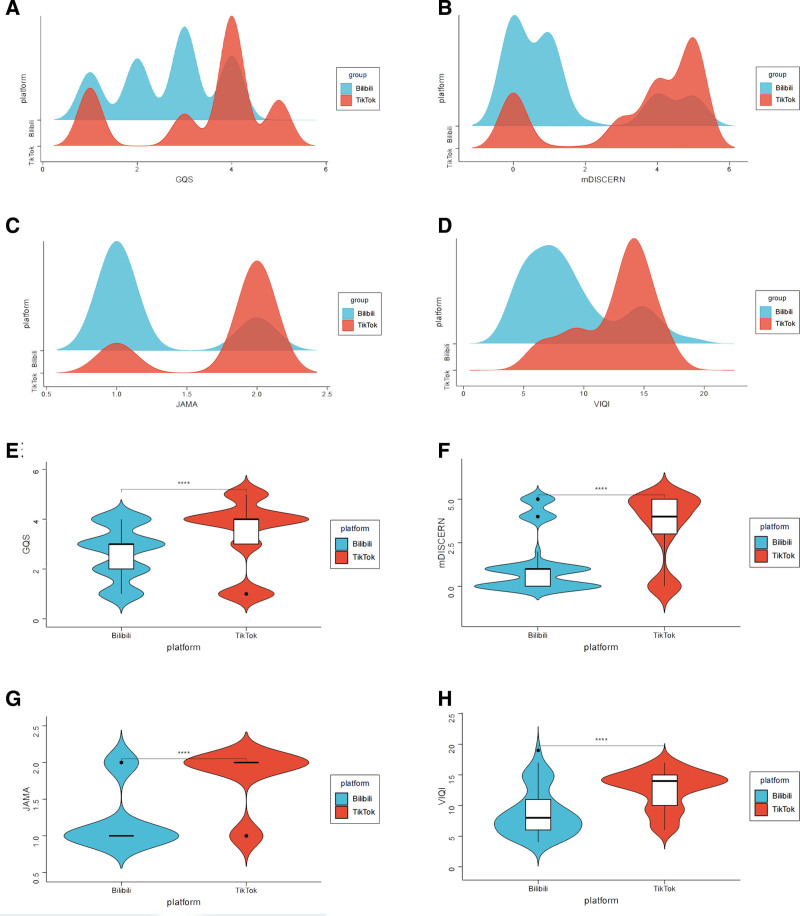
Comparison of information quality and reliability scores between TikTok and Bilibili. (A–D) Density distributions of GQS, mDISCERN, JAMA, and VIQI scores by platform. (E–H) Violin plots with embedded boxplots comparing GQS, mDISCERN, JAMA, and VIQI scores between TikTok and Bilibili. GQS = Global Quality Score, JAMA = Journal of the American Medical Association, mDISCERN = modified DISCERN, VIQI = Video Information and Quality Index.

Douyin videos scored significantly higher than Bilibili videos on all 4 evaluation tools: GQS, mDISCERN, JAMA, and VIQI (all *P* < .001; Table 2). Distribution plots showed the same pattern, with Douyin videos shifted toward higher score ranges and Bilibili videos concentrated at lower levels (Fig. 5). Thus, although Bilibili videos were usually longer and sometimes generated more interaction, Douyin videos showed better educational usefulness, source transparency, and overall presentation quality.

### 6.5. Quality and reliability by uploader type

**Figure 6.**
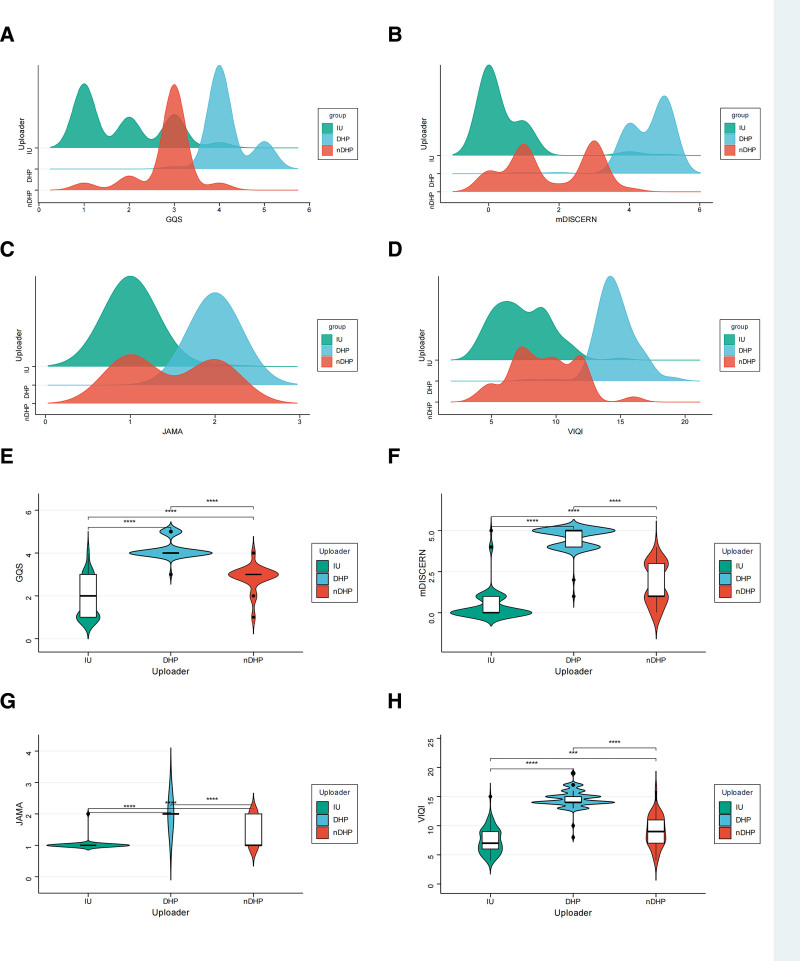
Comparison of information quality and reliability scores across uploader types. (A–D) Density distributions of GQS, mDISCERN, JAMA, and VIQI scores among different uploader types. (E–H) Violin plots with embedded boxplots comparing GQS, mDISCERN, JAMA, and VIQI scores among IU, DHP, and nDHP. DHPs = doctors or other health professionals, GQS = Global Quality Score, IU = individual users, JAMA = Journal of the American Medical Association, mDISCERN = modified DISCERN, NDHPs = non-doctor health professionals or health-related organization, VIQI = Video Information and Quality Index.

**Figure 7.**
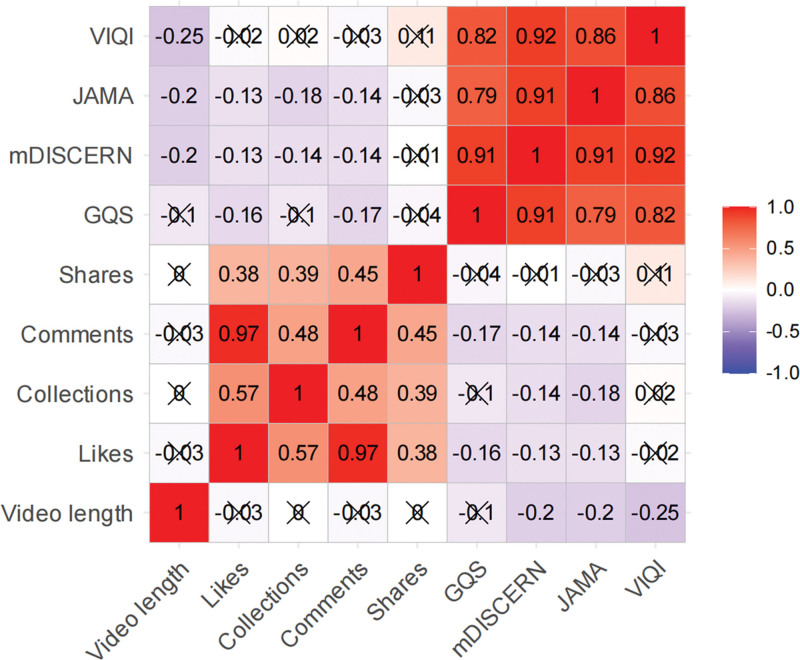
Correlation heatmap of video characteristics, engagement metrics, and quality scores. The heatmap presents pairwise correlation coefficients among video length, likes, collections, comments, shares, and the 4 evaluation scores (GQS, mDISCERN, JAMA, and VIQI). Engagement indicators were strongly correlated with one another, whereas their correlations with quality and reliability scores were generally weak. Cross marks indicate nonsignificant correlations. GQS = Global Quality Score, JAMA = Journal of the American Medical Association, mDISCERN = modified DISCERN, VIQI = Video Information and Quality Index.

When videos were grouped by uploader type, DHP videos consistently achieved the highest scores on GQS, mDISCERN, JAMA, and VIQI (all *P* < .001; Table 3). Videos by non-doctor health professionals or health-related organizations generally ranked between DHP and IU videos. IU videos showed the lowest quality and reliability but the strongest dissemination performance. This gradient was consistent across the distribution plots and combined comparison figures (Figs. 6 and 7).

### 6.6. Correlation analysis

The 4 quality evaluation tools were strongly positively correlated with one another (*r* = 0.79–0.92), supporting internal consistency of the scoring framework. Engagement metrics were also moderately to strongly correlated, especially likes and comments. In contrast, associations between engagement indicators and quality scores were weak or negligible. Video duration showed only weak relationships with engagement and weak negative relationships with quality-related indicators (Fig. 7). These findings indicate that neither popularity nor longer duration can be used as a dependable proxy for educational quality.

## 7. Discussion

This cross-sectional content analysis showed that educational videos on cognitive impairment available on Douyin and Bilibili were highly variable in quality and reliability and had notable gaps in thematic coverage. Across both platforms, symptoms and treatment were emphasized, whereas epidemiology, diagnosis, and prognosis were frequently neglected. Douyin videos achieved better quality and reliability scores, while videos uploaded by IUs generated stronger engagement. These findings are broadly consistent with previous studies showing uneven quality of health information on social media video platforms.^[8,15]^

A key issue raised by the comparative results is that Douyin and Bilibili are not functionally identical platforms. Douyin is designed around short, rapidly consumed videos with strong algorithmic distribution, concise message framing, and high-frequency exposure. Bilibili, by contrast, typically supports longer-form videos, more extended narration, and a community culture that can encourage deeper engagement through collections and sharing. These structural differences likely influenced both the observed dissemination patterns and the educational profiles of the included videos. The superior quality scores observed on Douyin should therefore not be interpreted as evidence that shorter videos are inherently better, but rather that concise, tightly structured, professionally produced content may perform well under short-video conditions.

Conversely, the longer duration of Bilibili videos did not automatically translate into better educational quality or reliability. Longer videos may provide more room for explanation, but they may also include personal narrative, unsystematic commentary, or loosely organized information. This helps explain why Bilibili videos generated stronger collections and shares yet still scored lower on GQS, mDISCERN, JAMA, and VIQI. In other words, platform affordances shape not only how content is consumed but also how health information is produced and presented.

The imbalance in content structure is clinically meaningful. A focus on symptoms alone may help viewers recognize possible warning signs, but insufficient discussion of epidemiology, diagnosis, and prognosis may limit understanding of who is at risk, when formal evaluation is needed, and why early management matters. For conditions such as cognitive impairment, where delayed recognition can affect access to assessment, care planning, and long-term support, incomplete educational content may reduce the practical value of otherwise popular videos.^[9,10]^

Uploader identity also played a major role. Videos produced by doctors and other health professionals showed consistently higher quality and reliability scores, whereas IU videos were more successful in attracting attention.^[16,17]^ This pattern suggests a persistent tension between scientific rigor and platform-native communication styles. Professionally generated content may be more accurate and better sourced, but it may not always be packaged in a way that maximizes audience engagement. Future digital health strategies should therefore encourage collaboration between clinicians, communication specialists, and platforms to improve both accuracy and reach.^[18,19]^

The weak correlations between engagement metrics and quality scores further underscore that visibility should not be equated with educational merit. Likes, shares, comments, and collections capture user response, but they do not reliably identify scientifically sound material.^[20,21]^ For viewers, this means that highly circulated videos should still be interpreted cautiously. For platforms, it suggests a role for stronger source labeling, credibility cues, and recommendation mechanisms that do not rely exclusively on popularity-based signals.

This study also highlights a methodological challenge in evaluating short-video health information. GQS, mDISCERN, JAMA, and VIQI remain useful because they are widely recognized and allow comparison with prior studies.^[8,9]^ More broadly, social media is increasingly used in dementia care and for health purposes overall, reinforcing the need for stronger quality governance in platform-based education.^[22,23]^ Similar concerns regarding variable reliability on video platforms have been reported in disease-specific analyses of thyroid cancer and deep brain stimulation content.^[24,25]^ Comparable issues have also been described in studies of occupational therapy and prostate cancer videos.^[26,27]^ Related patterns have been observed in ophthalmic analyses of keratoconus-related YouTube videos.^[28,29]^ Together, these findings suggest that the mismatch between visibility and scientific quality is not unique to cognitive impairment.^[30]^ However, they were not specifically designed for short-video ecosystems. We therefore added explicit short-video-oriented operational rules to improve scoring consistency. Even so, some constructs such as citation quality, disclosure, and completeness remain difficult to evaluate when information is embedded in voice-over, captions, profile pages, or linked descriptions. Future work should consider developing or validating assessment tools specifically for brief, platform-native health videos.

This study has several limitations. First, only the first 150 search results from each platform at a single time point were included, so the sample may have been influenced by ranking algorithms and temporal fluctuations in visibility. Second, engagement metrics are dynamic and may change over time. Third, although we clarified platform-specific scoring procedures, the assessment tools used were not originally designed for short-video media. Fourth, the study focused on 2 Chinese-language platforms, and the findings may not be generalizable to other linguistic or regulatory settings. Longitudinal, multilingual, and multiplatform studies are needed to better understand how online video ecosystems influence public knowledge and care-seeking related to cognitive impairment.

## 8. Conclusions

Educational videos on cognitive impairment on Douyin and Bilibili showed moderate overall quality and reliability, substantial heterogeneity, and incomplete content coverage. Douyin videos achieved higher quality and reliability scores, whereas videos uploaded by health professionals performed best overall. However, stronger engagement was more common among videos uploaded by IUs, and neither popularity nor duration reliably reflected educational value. Increasing professional participation, improving source transparency, and strengthening platform-level governance may enhance the accuracy and usefulness of online video-based health education on cognitive impairment.

## Acknowledgments

The authors thank all the researchers involved in video screening, data extraction, and quality assessment for their contributions to this study. No professional writing assistance was used for the preparation of this manuscript.

## Author contributions

**Conceptualization:** Yuzhang Liang, Haiyan Song, Chunyan Chen.

**Data curation:** Yuzhang Liang, Yifeng Xie, Xiaoxuan Fan, Yike Liu, Ya Na, Jiamin Gao.

**Formal analysis:** Yuzhang Liang, Chunyan Chen.

**Investigation:** Yuzhang Liang, Yifeng Xie, Xiaoxuan Fan, Ya Na, Jiamin Gao, Ying Ao.

**Methodology:** Yuzhang Liang, Yifeng Xie, Haiyan Song, Xiaoxuan Fan, Chunyan Chen.

**Visualization:** Yuzhang Liang, Xiaoxuan Fan.

**Supervision:** Haiyan Song, Chunyan Chen.

**Validation:** Haiyan Song, Chunyan Chen.

**Project administration:** Chunyan Chen.

**Writing – original draft:** Yuzhang Liang.

**Writing – review & editing:** Yuzhang Liang, Haiyan Song, Chunyan Chen.








