# Supplementary material for: Reliability and quality of cognitive impairment educational content on Douyin and Bilibili: A cross-sectional content analysis
Source: Medicine (Baltimore). 2026 May 22;105(21):e48941. doi: 10.1097/MD.0000000000048941 (PMC13201003; doi:10.1097/MD.0000000000048941)
Supplement: Supplementary file 1 [file medi-105-e48941-s001.doc]

Table S1. Global Quality Score criteria.

| **Description** | **Score** |
| --- | --- |
| Poor quality; poor flow of the videos; most information missing; not at all useful for patients | 1 |
| Generally poor quality; some information listed, but many important topics missing; of very limited use to patients | 2 |
| Moderate quality; suboptimal flow; some important adequately discussed, but other information poorly discussed; somewhat useful for patients | 3 |
| Good quality and generally good flow; most of the relevant information listed, but some topics not covered; useful for patients | 4 |
| Excellent quality and flow; very useful for patients | 5 |

Abbreviations: GQS, Global Quality Score.
