# Supplementary material for: Reliability and quality of cognitive impairment educational content on Douyin and Bilibili: A cross-sectional content analysis
Source: Medicine (Baltimore). 2026 May 22;105(21):e48941. doi: 10.1097/MD.0000000000048941 (PMC13201003; doi:10.1097/MD.0000000000048941)
Supplement: Supplementary file 2 [file medi-105-e48941-s002.doc]

**Table S2. Modified DISCERN criteria.**

| **Reliability Score** |
| --- |
| 1. Is the video clear, concise, and understandable? |
| 2. Are valid sources cited? |
| 3. Is the content presented balanced and unbiased? |
| 4. Are additional sources of content listed for patient reference? |
| 5. Are areas of uncertainty mentioned? |

Abbreviations: mDISCERN, modified DISCERN.
